# The *in vitro* activity of iron chelator deferiprone against *Candida (Candidozyma) auris* in combination with antifungal agents

**DOI:** 10.1093/mmy/myaf116

**Published:** 2025-12-13

**Authors:** Zoltán Tóth, Bálint Farkas, László Majoros, Ágnes Jakab, Andrew M Borman, István Varga, Petra Rita Tóth, Renátó Kovács

**Affiliations:** Department of Medical Microbiology, Faculty of Medicine, University of Debrecen, Debrecen 4032, Hungary; Department of Medical Microbiology, Faculty of Medicine, University of Debrecen, Debrecen 4032, Hungary; Doctoral School of Pharmaceutical Sciences, University of Debrecen, Debrecen 4032, Hungary; Department of Medical Microbiology, Faculty of Medicine, University of Debrecen, Debrecen 4032, Hungary; Department of Medical Microbiology, Faculty of Medicine, University of Debrecen, Debrecen 4032, Hungary; UK National Mycology Reference Laboratory, UK Health Security Agency, Science Quarter, Southmead Hospital, Bristol BS10 5NB, UK; Medical Research Council Centre for Medical Mycology (MRC CMM), University of Exeter, Exeter EX4 4QD, UK; Non-Independent Department of Periodontology, Faculty of Dentistry, University of Debrecen, Debrecene, 4032, Hungary; Department of Dentistry, University of Debrecen Clinical Centre, University of Debrecen, Debrecen, 4032, Hungary; Department of Medical Microbiology, Faculty of Medicine, University of Debrecen, Debrecen 4032, Hungary

**Keywords:** *Candida auris*, deferiprone, iron, synergy, echinocandin resistant, chequerboard assay

## Abstract

*Candida (Candidozyma) auris* is well known for its limited susceptibility to conventional antifungal agents, underscoring the need for novel alternative therapeutic approaches. One such approach, which involves restricting the availability of micronutrients—including iron—is a promising antimicrobial strategy and proven to enhance the efficacy of various antimicrobial agents *in vitro*. In this study, we evaluated the activity of deferiprone, a clinically approved iron-chelating agent, in combination with echinocandins, amphotericin B and fluconazole against *C. auris* strains, including three echinocandin-resistant isolates representing the four major clades. Drug–drug interactions were assessed using the chequerboard methodology. Fractional inhibitory concentration index scores ranged from 0.5 to 2.25 and 0.1875 to 4.125 for anidulafungin; 0.375 to 1.5 and 0.125 to 1.5 for micafungin; 0.3125 to 1.5 and 0.3125 to 2 for caspofungin; 0.375 to 2.0625 and 0.375 to 2 for amphotericin B; and 0.625 to 2 and 0.625 to 2 for fluconazole at 24 and 48 h, respectively. Synergistic interactions were most frequently observed with echinocandins, while interactions with fluconazole and amphotericin B were generally additive or indifferent, except in Clade II. Antagonism was observed in only one instance. These findings suggest that iron chelation may potentiate the antifungal activity of echinocandins against *C. auris* in a clade- and isolate-specific manner.

## Introduction

Iron is an essential micronutrient for both human cells and pathogens.^1^ In this context, it has been identified as a promising anti-infective target, and numerous studies have demonstrated that limiting iron availability can significantly enhance the activity of conventional antimicrobials against various bacterial and fungal pathogens, in both planktonic and sessile populations.^2–4^ For such repurposing strategies, clinically available iron chelators—such as desferrioxamine, deferiprone, and deferasirox—originally indicated for the amelioration of iron overload (e.g., in beta-thalassemic patients),^5^ seem especially suitable due to their longstanding clinical use. Deferiprone was the second iron-chelating drug to be introduced and represented a significant improvement over desferrioxamine owing to its oral route of administration. Furthermore, in contrast to desferrioxamine, it does not act as xenosiderophore for specific pathogens and therefore does not increase the risk of infectious etiologies.^5,6^ While the clinically available iron chelators deferiprone and deferasirox exhibit profound activity against *Rhizopus oryzae* and *Cryptococcus neoformans*, the clinically relevant *Candida* species appear to be less susceptible, although iron limitation by various compounds has been found to synergize with fluconazole activity against them.^7–11^ However, data on the emerging pathogen *Candida (Candidozyma) auris* are completely lacking in this regard.

*Candida auris* is recognized as a highly resistant and tolerant species to conventional antifungal agents, as reflected by its designation by the World Health Organization as a critical priority fungal pathogen, calling for increased research efforts and the development of novel therapeutic strategies.^12,13^ This species is also known to pose a significant threat to patients with hematologic malignancies.^14–16^ This patient population frequently receives blood transfusions and parenteral iron therapy, often leading to secondary iron overload—a condition associated with enhanced fungal virulence.^7,17^ Various *Candida* species have been shown to effectively regulate iron acquisition genes, enabling them to efficiently adapt to both iron-poor and iron-rich environments.^18,19^ Moreover, previous studies have shown that increased iron availability may promote *Candida* persistence and pathogenicity,^11,20,21^ supporting the rationale for therapeutic iron restriction. Given that iron plays a crucial role in various stress responses of *C. albicans*^22^ and that iron starvation induces several metabolic changes in both *C. albicans* and *C. auris*,^23,24^ we aimed to investigate the potential of iron limitation via a clinically available iron chelator as a novel therapeutic strategy. We examined whether deferiprone-mediated iron chelation can suppress the growth of *C. auris* and enhance the efficacy of traditional antifungals. In addition, we assessed whether clade-specific differences influence the response to iron chelation and antifungal combinations.

## Materials and methods

### Origin of the isolates

The tested isolates belonged to the prevalent clades of I–IV, including the reference strain (Isolate 209 = NCPF 13029 = CBS 10913) (Table 1), and were obtained from the National Mycology Reference Laboratory, UK. For each clade, five isolates were tested except for Clade II, which included four isolates. Sequencing of the echinocandin-resistant isolates was performed in our previous study.^25^ Isolates were re-identified prior to experiments using a Bruker Microflex LT instrument in conjunction with Compass 4.1 software (Bruker Daltonics, Bremen, Germany).

**Table 1.** MIC values of anidulafungin (ANF), caspofungin (CSF), micafungin (MCF), amphotericin B (AMB), fluconazole (FLU), and deferiprone (DEF) against *Candida auris* isolates from the four major clades at 24 and 48 h.

Isolate	Clade	FKS	ANF MIC 24 h	ANF MIC 48 h	MCF MIC 24 h	MCF MIC 48 h	CSF MIC 24 h	CSF MIC 48h	FLU MIC 24h	FLU MIC 48h	AMB MIC 24h	AMB MIC 48h	DEF MIC50 24h	DEF MICtotal 24h	DEF MIC50 48h	DEF MIC total 48 h
**10**	I	NA	0.125	0.125	0.125	0.25	0.25	0.25	32	32	1	2	64	128	128	128
**20**	I	HS2 R1354H	1	8	1	4	32	32	64	128	1	1	32-64	64	64	64
**27**	I	NA	0.06-0.12	0.125	0.125	0.25	0.125	0.25	32	32	1	2	64	128	128	128
**28**	I	HS1 S639Y	16	32	8	32	16	32	16	32	1	2	16-32	128	128	128
**208**	I	HS1 S639P	8	32	16	32	32	32	32	128	1	2	64	128	128	128
**15**	II	NA	0.06	0.06	0.125	0.25	0.125	0.125	128	128	0.5	1	32	64	64	128
**209**	II	NA	0.03	0.06	0.03	0.06	0.06	0.06	2	4	0.5	0.5	16	32-64	32	64
**12 372**	II	NA	0.06	0.06	0.125	0.125	0.125	0.125	128	128	0.5	0.5	32	64	64	128
**12 373**	II	NA	0.03-0.06	0.03-0.06	0.125	0.125	0.06	0.06	64	128	0.5	1	16	64	64	128
**2**	III	NA	0.125	0.25	0.25	0.25-0.5	0.25	0.25	32	64	1	2	32	64	64	64
**185**	III	NA	0.06	0.06	0.125	0.125	0.125	0.125	128	128	1	1	128	256	256	512
**204**	III	NA	0.06	0.125	0.06	0.125	0.125	0.125	128	128	0.5	0.5	64	256	256	512
**206**	III	NA	0.125	0.125	0.125	0.25	0.125	0.125	128	128	0.5	1	64	64	64	128
**228**	III	NA	0.06	0.06	0.06	0.125	0.125	0.25	128	128	1	1	64	128	128	128
**I-24**	IV	NA	0.06	0.125	0.125	0.25	0.125	0.125	32	64	0.5	1	64	128	128	128
**I-156**	IV	NA	0.03-0.06	0.06	0.06	0.125	0.125	0.125	32-64	64	0.5	1	32	128	128	128
**13 108**	IV	NA	0.03	0.06	0.06	0.06	0.06-0.125	0.125	64	128	0.5	1	64	128	128	256
**13 112**	IV	NA	0.06	0.25	0.125	0.25	0.125	0.25	64	128	0.5	1	64	128	128	128
**16 565**	IV	NA	0.06	0.06	0.125	0.125	0.125	0.25	2	4	0.5	0.5	32	64-128	128	128

Notes: MICs were determined in duplicate according to the CLSI M27-A3 protocol. Isolate 209 = NCPF 13029 = CBS 10913.

### Determination of Minimal Inhibitory Concentrations

Minimal Inhibitory Concentration (MIC) values were determined according to the CLSI M27-A3 protocol in RPMI-1640 supplemented with 0.2% glucose, without L-glutamine in U-bottom, tissue-treated plates (TPP, Techno Plastic Products AG, Trasadingen, Switzerland).^26^ The tested concentration range was 0.004–2 mg/l for amphotericin B and echinocandins (caspofungin, anidulafungin, micafungin) for susceptible isolates and 0.032–32 mg/l for echinocandin resistant ones. For fluconazole, a concentration range of 0.25–128 mg/l was tested, and for deferiprone, 1–512 mg/l. RPMI-1640 was obtained from Capricorn Scientific (Ebsdorfergrund, Germany). Antifungals and deferiprone were acquired from Merck (Budapest, Hungary). MIC determination was performed in duplicate on separate days. Since no standardized endpoints are available for deferiprone, both 50% and complete inhibition endpoints were reported. The tested concentration ranges were determined based on preliminary experiments.

### Checkerboard microdilution experimental setting

The interaction between deferiprone and antifungal agents was assessed using a modified CLSI M27-A3-based checkerboard method after 24 and 48 h of static incubation at 35°C.^27^ Experiments were performed in F-bottom, tissue-culture-treated TPP plates, and optical density at 600 nm (OD_600_) was measured at both time points using a Multiskan Sky plate reader (Thermo Fisher Scientific, Waltham, MA, USA). OD values were obtained using Thermo Fisher’s SkanIt RE software (version: 6.0.2), with the blank subtraction option enabled. The vendors for antifungal agents and RPMI-1640 were the same as those used in the MIC determination experiments. The tested concentration ranges were as follows: 0.008–2 mg/l for amphotericin B; 0.004–1 mg/l for echinocandins in susceptible; 0.125–32 mg/l for echinocandin resistant isolates; 0.25–64 mg/l for fluconazole; and 2–128 mg/l for deferiprone. The concentration ranges were determined based on the MIC values obtained during the susceptibility testing experiments.

### Determination of fractional inhibitory concentration index and Bliss average scores

Fractional MICs were calculated as follows: ΣFIC = FIC_A_ + FIC_B_ = MIC_A_^comb^/MIC_A_^alone^ + MIC_B_^comb^/MIC_B_^alone^, where MIC_A_^alone^ and MIC_B_^alone^ stand for MICs of drugs A and B alone, and MIC_A_^comb^ and MIC_B_^comb^ are the MIC values of drugs A and B in combination at isoeffective combinations. Fractional Inhibitory Concentration Index (FICI) was defined as the lowest ΣFIC observed. A 50% endpoint was applied for echinocandins and fluconazole alone and for their respective isoeffective concentrations, while for amphotericin B, total reduction (> 99%) was used. For deferiprone, the same endpoint was used as for the respective antifungal: 50% growth reduction for fluconazole and the echinocandins, and complete inhibition for amphotericin B experiments. If the MIC value was higher than the highest tested drug concentration, the next highest twofold concentration was considered as MIC. A FICI value of ≤ 0.5 was interpreted as synergistic, > 0.5 to ≤ 1 as additive, > 1 to ≤ 4 as indifferent, and > 4 as antagonistic.^28^

Interactions were further evaluated using the Bliss independence model with the SynergyFinder 3.0 webtool.^29^ The OD_600_ values were converted to percentages, representing the relative growth compared to the control. Complete matrices were then uploaded, and Bliss average scores were acquired with Readout: Viability, Calculate Synergy: ON, Method: Bliss, Correction: ON, Visualize synergy scores: ON options. The obtained Bliss average scores were visualized with GraphPad Prism 8.0.1 (GraphPad Software, Boston, MA, United States). Interactions were interpreted as synergistic, additive, or antagonistic when the Bliss average score was ≥ 10, between < 10 and > –10, and ≤ –10, respectively.^29,30^

## Results

According to the tentative Centers for Disease Control and Prevention breakpoints, 16 out of 19 isolates were susceptible to echinocandins, 3 to fluconazole, and all isolates were susceptible to amphotericin B, based on the available MIC breakpoint of ≤ 2 mg/l (Table 1).^31^ Deferiprone showed *in vitro* activity, with partial and complete inhibitory concentrations ranging from 16 to 256 mg/l and 32 to 256 mg/l, respectively, after 24 h. At 48 h, these ranges shifted to 32–256 mg/l and 64–512 mg/l for partial and total inhibition, respectively. Notably, the MIC values of the tested antifungals increased 2- to 4-fold after 48 h in some cases.

**Figure 1.**
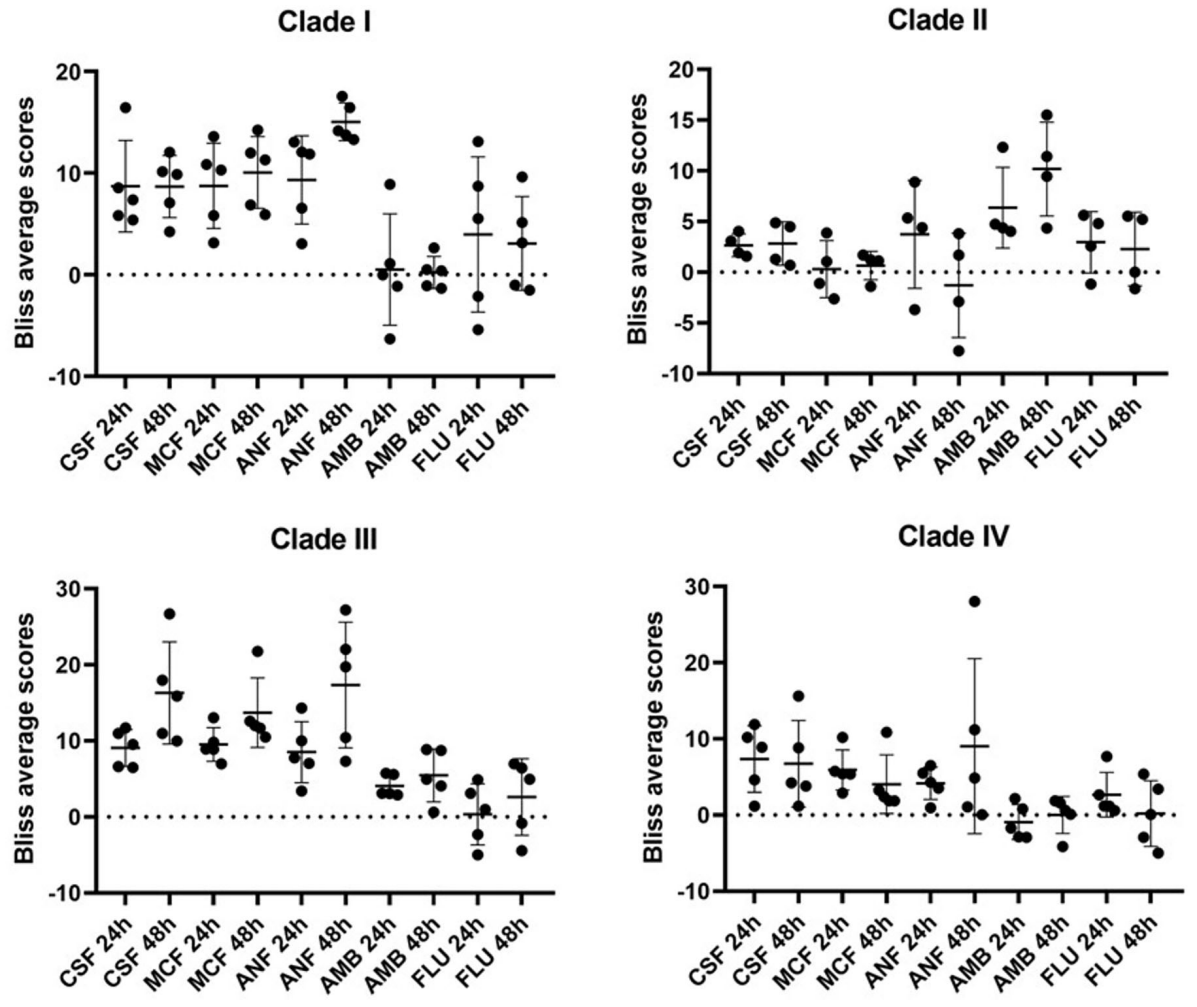
Bliss average scores at 24 and 48 h for combinations of anidulafungin (ANF), caspofungin (CSF), micafungin (MCF), amphotericin B (AMB), and fluconazole (FLU) with deferiprone (DEF) across different *Candida auris* clades. Error bars represent mean ± standard deviation (SD). Individual Bliss average scores are provided in Supplementary Table 1.

Echinocandins exhibited predominantly positive interaction with deferiprone against Clade I, III, and IV isolates—including echinocandin-resistant strains—with FICI values ranging from 0.3125 to 1.5 after 24 h and from 0.125 to 1 after 48 h. Synergy (FICI ≤ 0.5) was observed in 17 echinocandin/isolate combinations after 24 h and in 22 combinations after 48 h, including echinocandin-resistant isolates. Against Clade I isolates, anidulafungin produced the highest number of synergistic interactions at both 24 h and 48 h, as determined by FICI (3 and 4 out of 5 respectively). According to Bliss average scores, three interactions were also synergistic at 24 h, and all five reached synergy at 48 h. In contrast, micafungin and caspofungin demonstrated less consistent synergistic activity. For Clade III isolates, micafungin yielded the greatest number of synergistic interactions (1 out of 5 at 24 h and all 5 at 48 h). This was well reflected by the Bliss average scores, which exceeded the threshold of 10 for only one isolate at 24 h, but for all isolates at 48 h. Clade IV isolates originating from South America exhibited notably weaker interactions (Table 2). Synergistic effects were most frequently observed for anidulafungin and micafungin at both time points according to FICI. Bliss average scores reflected the same trend, ranging from 0.948 to 16.445 after 24 h and from 0.058 to 28.035 after 48 h (Fig. 1). However, the synergy threshold was exceeded in only a few cases. Discrepant results—such as synergy detected at 24 h but not at 48 h and vice versa—were observed in nine deferiprone–echinocandin combinations for Clade I, seven for Clade III, and five for Clade IV according to FICI, and in seven, eight and three combinations, respectively, according to Bliss average scores. Among these, three represented positive changes (i.e., synergy achieved at 48 h) for Clade I by both FICI and Bliss analysis. For Clade III isolates, positive changes were observed in seven and eight combinations by FICI and Bliss average scores respectively. For Clade IV, synergy emerged at 48 h in three cases according to FICI and in one case according to Bliss analysis. Additionally, additive interactions between echinocandins and deferiprone were observed in 35 and 28 combinations at 24 and 48 h, respectively according to FICI.

**Table 2.** Proportions of synergistic (SYN), additive (ADD), indifferent (IND), and antagonistic (ANT) interactions based on FICI scores for combinations of anidulafungin (ANF), caspofungin (CSF), micafungin (MCF), amphotericin B (AMB), and fluconazole (FLU) with deferiprone (DEF) at 24 and 48 h across different *Candida auris* clades.

	ANF 24h	ANF 48h	CSF 24 h	CSF 48h	MCF 24h	MCF 48h	AMB 24h	AMB 48h	FLU 24h	FLU 48h
**Clade I**	SYN 3/5	SYN 4/5	SYN 4/5	SYN 0/5	SYN 3/5	SYN 3/5	SYN 1/5	SYN 0/5	SYN 0/5	SYN 0/5
	ADD 2/5	ADD 1/5	ADD 1/5	ADD 5/5	ADD 2/5	ADD 2/5	ADD 0/5	ADD 2/5	ADD 5/5	ADD 5/5
	IND 0/5	IND 0/5	IND 0/5	IND 0/5	IND 0/5	IND 0/5	IND 4/5	IND 3/5	IND 0/5	IND 0/5
	ANT 0/5	ANT 0/5	ANT 0/5	ANT 0/5	ANT 0/5	ANT 0/5	ANT 0/5	ANT 0/5	ANT 0/5	ANT 0/5
**Clade II**	SYN 0/4	SYN 0/4	SYN 0/4	SYN 0/4	SYN 0/4	SYN 0/4	SYN 4/4	SYN 4/4	SYN 0/4	SYN 0/4
	ADD 3/4	ADD 2/4	ADD 2/4	ADD 0/4	ADD 3/4	ADD 3/4	ADD 0/4	ADD 0/4	ADD 4/4	ADD 4/4
	IND 1/4	IND 1/4	IND 2/4	IND 4/4	IND 1/4	IND 1/4	IND 0/4	IND 0/4	IND 0/4	IND 0/4
	ANT 0/4	ANT 1/4	ANT 0/4	ANT 0/4	ANT 0/4	ANT 0/4	ANT 0/4	ANT 0/4	ANT 0/4	ANT 0/4
**Clade III**	SYN 2/5	SYN 3/5	SYN 0/5	SYN 2/5	SYN 1/5	SYN 5/5	SYN 1/5	SYN 0/5	SYN 0/5	SYN 0/5
	ADD 3/5	ADD 2/5	ADD 5/5	ADD 3/5	ADD 4/5	ADD 0/5	ADD 4/5	ADD 3/5	ADD 1/5	ADD 2/5
	IND 0/5	IND 0/5	IND 0/5	IND 0/5	IND 0/5	IND 0/5	IND 0/5	IND 2/5	IND 4/5	IND 3/5
	ANT 0/5	ANT 0/5	ANT 0/5	ANT 0/5	ANT 0/5	ANT 0/5	ANT 0/5	ANT 0/5	ANT 0/5	ANT 0/5
**Clade IV**	SYN 1/5	SYN 2/5	SYN 2/5	SYN 2/5	SYN 1/5	SYN 1/5	SYN 0/5	SYN 0/5	SYN 0/5	SYN 0/5
	ADD 3/5	ADD 3/5	ADD 3/5	ADD 3/5	ADD 4/5	ADD 4/5	ADD 1/5	ADD 1/5	ADD 4/5	ADD 5/5
	IND 1/5	IND 0/5	IND 0/5	IND 0/5	IND 0/5	IND 0/5	IND 4/5	IND 4/5	IND 1/5	IND 0/5
	ANT 0/5	ANT 0/5	ANT 0/5	ANT 0/5	ANT 0/5	ANT 0/5	ANT 0/5	ANT 0/5	ANT 0/5	ANT 0/5

Note: Individual FICI scores are provided in Supplementary Table 1.

In contrast to Clades I, III, and IV, Clade II isolates behaved differently. They showed no synergistic interaction with echinocandins and isolate 15 even exhibited antagonism with anidulafungin (FICI = 4.125; Bliss score = –7.76 at 48 h). Interestingly, Clade II isolates demonstrated a marked synergistic interaction with amphotericin B—an effect uncommon in other clades—with FICI values of 0.5 for all isolates at 24 h, and 0.375–0.5 at 48 h (Table 2). Fluconazole generally showed no significant interaction with deferiprone at the tested concentrations and time points. FICI values ranged from 0.5 to 2 at 24 h and from 0.625 to 2 at 48 h, indicating predominantly additive to indifferent interactions (Table 2). A Bliss average score exceeding 10 was observed for one isolate at 24 h, but not at 48 h (Fig. 1).

## Discussion

Our results suggest that iron chelation with deferiprone may enhance the activity of echinocandins and in a clade-specific manner, amphotericin B against *Candida auris*. In a comprehensive study, Simm *et al*. (2022) demonstrated that iron chelation with pyrvinium pamoate led to downregulation of *FKS1* and chitin synthases, genes essential for cell wall biosynthesis.^24,32^ It remains to be determined whether deferiprone exerts a similar effect; however, iron chelation may also indirectly affect other cellular stress pathways, mitochondrial function, or iron-dependent enzymes. Such changes might sensitize *C. auris* to echinocandins in a manner that is both clade- and strain-dependent. Our findings are partly consistent with the observations of Preeti and Pasrija (2025), who recently reported synergistic interactions between echinocandins and the iron chelators deferiprone and deferoxamine against Clade II and Clade III *C. auris* isolates, but not against those belonging to Clades I and IV.^33^ These differences may reflect methodological variations (e.g., inhibition endpoints, drug concentrations, and incubation times), as well as strain-level differences within clades. Comparative studies using shared reference isolates and genomic characterization (e.g., *FKS1* or *FKS2* mutations) could help clarify these discrepancies.

The near-complete absence of significant interaction with fluconazole was somewhat unexpected. Previous reports have indicated that iron chelation by various agents can increase the susceptibility of fluconazole-resistant *Candida* isolates to fluconazole.^10,34^ Although the highest fluconazole concentration tested in our experimental setting was only 64 mg/l, no synergy was observed in either fluconazole-susceptible or -resistant isolates. Similarly, Simm *et al*. (2022) previously reported additive, but not synergistic effects of fluconazole combined with pyrvinium pamoate against *C. auris*.^24^ In contrast, Zhang *et al*. (2025) found that pyrvinium pamoate synergized with other azoles—posaconazole, voriconazole, and itraconazole—although combinations with fluconazole against *C. auris* were not examined in that study.^35^ Interestingly, aprepitant, an antiemetic agent, has been shown to act synergistically with fluconazole, voriconazole, and itraconazole via iron chelation mechanisms.^36^ These findings suggest that iron chelators may differ in their potentiating effects and that antifungal agents with similar mechanisms of action can exhibit distinct interaction profiles with different chelators against *C. auris*.

To the best of our knowledge, this is the first study to examine the interaction between amphotericin B and an iron chelator against *C. auris*. Although direct comparison with previous studies is not possible, the clade-specific response observed here may warrant further investigation. Iron starvation has been reported to increase membrane fluidity and decrease cellular ergosterol levels in *C. albicans*.^37^ Reduced ergosterol content in the cell membrane is a well-known mechanism of amphotericin B resistance.^38^ Nevertheless, combinations of amphotericin B with iron chelator deferasirox have demonstrated synergistic interaction *in vivo* against *Aspergillus fumigatus*.^39^

The possibility arises that iron deficiency may also influence the *in vivo* efficacy of antifungal agents. In our previous work, we observed increased fungicidal activity of echinocandins against *C. auris* isolates in the presence of human serum,^40^ particularly among Clades III and IV isolates originating from Israel.^40^ Interestingly, *in vitro* activity often underestimates the *in vivo* efficacy of echinocandins against *C. auris*.^40,41^ This discrepancy may, at least in part, result from the highly restricted availability of free iron *in vivo*.^42,43^ We therefore hypothesize that the potentiation of echinocandin activity under iron-limited conditions—whether through host-mediated sequestration or pharmacological chelation—may contribute to explaining this paradox. Additionally, in our previous study, we observed an unusually good therapeutic response to amphotericin B in a neutropenic murine model infected with Clade II isolates, whereas isolates from other clades responded poorly.^44^ The observed synergy between amphotericin B and deferiprone in Clade II may provide a possible explanation—namely, that iron starvation enhances susceptibility to amphotericin B in these isolates.

The substantial differences in virulence observed *in vivo* among the various *C. auris* clades cannot be overlooked when interpreting these results. We did not detect significant differences in deferiprone MIC values among the tested clades, even though Clades I and especially IV exhibit markedly higher virulence in different animal models.^45,46^ Given that, in microbial pathogens more efficient iron acquisition is often associated with increased virulence,^47^ it is encouraging that deferiprone exhibits clade-independent activity.

Although combination therapy with deferiprone appears promising *in vitro*, several limitations temper its clinical potential. Although *in vitro* cytotoxicity assays demonstrated limited cell toxicity—up to 10 mM in fibroblast cells^48^—the relatively high MIC values, short plasma half-life and moderate peak serum concentration suggest that therapeutically relevant concentrations may not be achievable *in vivo*.^17^ This is reminiscent of clinical experiences with deferasirox, which showed strong efficacy in animal models of mucormycosis, but failed to outperform standard treatments in human trials.^49,50^ An additional potential limitation in translating these findings to *in vivo* systems is that alternative iron acquisition mechanisms may compensate for the effects of deferiprone. While RPMI-1640 contains only inorganic iron, *C. auris* is capable of utilizing haem and haemoglobin, which are abundant *in vivo*, particularly within erythrocytes.^34^ However, how efficiently *C. auris* acquires haem remains to be elucidated, as it lacks candidalysin orthologs, considered the primary means of erythrocyte lysis in *C. albicans*.^34^

Nevertheless, novel iron chelators with improved antifungal profiles in the experimental phase, such as DIBI or NR-6226C, which exhibit enhanced activity against *Candida* species, could merit further evaluation.^10,51^ Furthermore, future studies using iron-restricted animal models are needed to better mimic various host conditions and assess the potential for therapeutic synergy.

## Supplementary Material

myaf116_Supplemental_File

## Data Availability

The data underlying this article are available in the article and in its online supplementary material.
